# Progression of Arterial Stiffness is Associated With Midlife Diastolic Blood Pressure and Transition to Late‐Life Hypertensive Phenotypes

**DOI:** 10.1161/JAHA.119.014547

**Published:** 2020-01-04

**Authors:** Alastair John Stewart Webb

**Affiliations:** ^1^ Wolfson Centre for Prevention of Stroke and Dementia University of Oxford United Kingdom

**Keywords:** arterial stiffness, hypertension, longitudinal cohort study, Hypertension

## Abstract

**Background:**

Hypertension‐associated cardiovascular events are particularly associated with elevated systolic blood pressure (SBP) in late life, yet long‐term interactions between SBP, diastolic BP (DBP) and arterial stiffness in development of late‐life hypertensive phenotypes remain unclear.

**Methods and Results:**

In the UK Biobank, we determined associations between arterial stiffness index (ASI), SBP, DBP, and their progression, and transition from normotension (<140/90 mm Hg) to hypertension or elevated ASI (>10 m/s). Associations were determined by general linear and logistic regression, adjusted for cardiovascular risk factors and variability of measurements across follow‐ups. Mean values of baseline SBP, DBP, and ASI were determined stratified by deciles of age, blood pressure, and ASI, with CIs determined by bootstrapping. In 169 742 participants at baseline, ASI was more strongly associated with DBP than SBP, before and after adjustment for risk factors (β: SBP, −0.01 [*P*<0.001]; DBP, 0.06 [*P*<0.001]), while DBP was more strongly associated with progression of ASI (n=13 761; β: SBP, 0.013 [*P*=0.01]; DBP, 0.038 [*P*<0.001]). Baseline ASI was associated with increasing SBP during follow‐up (β=0.02, *P*<0.001) but not DBP (β=0.0004, *P*=0.39), but was associated with a younger age of transition from rising to falling DBP (highest versus lowest quartile: 51.2; 95% CI, 49.9–52.3 versus 60.4; 95% CI, 59.6–61.3 [*P*<0.001]). ASI predicted the development of isolated systolic hypertension (odds ratio, 1.30; 95% CI, 1.22–1.39), particularly after adjustment for measurement variability (odds ratio, 2.29).

**Conclusions:**

Midlife DBP was the strongest predictor of progression of arterial stiffness, while arterial stiffness was associated with earlier transition to a falling DBP. Prevention of long‐term harms associated with arterial stiffness may require more intensive control of midlife DBP.

**Clinical Perspective**

**What Is New?**

- In a midlife, community‐based population, late‐life arterial stiffness and progression of arterial stiffness over ≈8 years were predicted by increased blood pressure (BP) in midlife, particularly for an elevated diastolic BP more than systolic BP.
- Increased arterial stiffness was associated with an earlier mean age of transition from a rising to a falling diastolic BP across the population, from 51 years in the top quartile of stiffness to 60 years in the bottom quartile.

**What Are the Clinical Implications?**

- Prevention of arterial stiffening and the associated transition to a late‐life hypertensive phenotype of falling diastolic BP is likely to depend on effective control of midlife diastolic BP in particular.

## Introduction

Systolic blood pressure (SBP) is the principal focus of modern control of hypertension because of the strong short‐ and mid‐term associations with acute vascular events,^1^ the proven efficacy of hypertensive treatment for isolated systolic hypertension^2^, ^3^, ^4^ and the greater population attributable burden of SBP‐associated cardiovascular events, reflecting the high incidence of both cardiovascular events and isolated systolic hypertension in the elderly.^5^ Specifically, SBP tends to increase and diastolic blood pressure (DBP) tends to decrease in late life.^6^ Therefore, short‐term associations with SBP in late life may not reflect the long‐term attributable risk of cardiovascular events caused by midlife diastolic hypertension, particularly if the risk associated with midlife DBP is mediated by late‐life SBP.

Arterial stiffness is associated with recurrent cardiovascular events and all‐cause mortality, independent of age, blood pressure (BP), and cardiovascular risk factors.^7^, ^8^ It is strongly associated with age and a history of hypertension,^9^ is often described as a measure of “vascular age,” and features in clinical guidelines as a marker of hypertensive end‐organ damage.^10^, ^11^ Studies that have assessed the longitudinal relationships between age, arterial stiffness, and BP have largely reported an association between increased SBP and progression of arterial stiffness,^9^, ^12^, ^13^ although some have reported no evidence of a direct relationship,^6^ or sex‐specific differences in longitudinal progression of arterial stiffness.^14^ However, studies have not reported the bidirectional interactions between increased SBP or DBP and progression of arterial stiffness and the modification of longitudinal changes in BP by increasing arterial stiffness in midlife.

In addition to being a marker of hypertensive end‐organ damage,^6^ arterial stiffness may directly induce end‐organ injury through reduced damping of the systolic pressure wave, increased pulse wave reflection from the periphery, and impaired Windkessel function of the aorta,^15^ resulting in increased systolic pressures, increased pulsatility of aortic BP, and greater transmission of pulsatile flow to the distal circulation.^16^ The low‐resistance arterial beds in the kidney and brain are particularly susceptible to the increased pulsatility of aortic blood flow, resulting in a strong association between aortic stiffness and cerebral arterial pulsatility with acute lacunar stroke,^17^ white matter injury,^18^ and the associated sequelae of decline in mobility, late‐onset refractory depression, and dementia.^19^ To develop strategies to prevent end‐organ injury, it is critical to understand the magnitude and direction of the relationship between BP and arterial stiffness, and the optimal period for intervention to prevent increased arterial stiffness.

The UK Biobank cohort is the largest, midlife community‐based cohort with measures of BP and arterial stiffness in nearly 170 000 participants.^20^ Furthermore, Biobank includes repeated measures in a large subset of the population, allowing estimation of the consistency of arterial stiffness and BP measures and the effect of variability in these measures on epidemiological associations over prolonged periods, providing a unique opportunity to understand the longitudinal interactions between arterial stiffness, SBP, and DBP. This allows estimation and adjustment of analyses for variability in measurements, determination of cross‐sectional associations at baseline using age as a surrogate for longitudinal changes in a large population, and validation of these associations by determining direct associations between baseline measures and within‐individual progression over time in a subgroup of the population. This also allows estimation of clinically relevant BP measures, including diagnosis of hypertension and median age for transition to late‐life phenotypes.

This study therefore aims to determine the relationship between midlife SBP or DBP and progression of arterial stiffness, and the converse relationship between arterial stiffness and longitudinal changes in BP.

## Methods

UK Biobank recruited >500 000 community‐based participants aged 40 to 69 years in 22 centers between 2006 and 2010.^20^ It includes baseline assessments of health status, lifestyle and environmental factors, cognitive status, and physical measures, including BP and arterial stiffness, with repeated assessments in 20 000 to 35 000 participants. Medical history of any diagnoses made by a doctor (diabetes mellitus, previous cardiovascular events, or other medical conditions) were self‐reported by participants at baseline and each follow‐up. All data used in these analyses are available from UK Biobank upon direct application by valid researchers and further materials can be obtained upon application to the corresponding author. UK Biobank was approved by the UK North West Multi‐centre Research Ethics Committee and all participants provided informed written consent.

Arterial stiffness index (ASI) in the UK Biobank study was measured using the PulseTrace PCA2 (CareFusion) (Field‐ID 21021)^21^ at baseline between 2009 and 2010, with repeated measures at a single center between 2012 and 2013 and at 3 centers between 2014 and 2019. The PulseTrace PCA2 uses finger photoplethysmography to obtain a pulse waveform during a 10‐ to 15‐second measurement. The measurement was repeated on a larger finger or on the thumb if less than two thirds of the waveform was visible or if the waveform did not stabilize within 1 minute. ASI is derived from the interval between the forward and presumed aortic‐reflected reverse traveling pulse wave, standardized to the standing height. SBP and DBP were measured twice at baseline and each follow‐up by a trained nurse after the participant had been at rest for at least 5 minutes in the seated position with a digital sphygmomanometer (Omron 705 IT; OMRON Healthcare Europe B.V.) with a suitably sized cuff. The average of the 2 SBP or DBP measurements was used in all analyses. Pulse pressure (PP) was calculated as SBP minus DBP and mean BP (MBP) as DBP plus one third of the PP.

Socioeconomic, health, lifestyle, and environmental factors were recorded at baseline through a self‐administered touchscreen questionnaire, supported by face‐to‐face interviews. All data were downloaded from UK Biobank, imported into R for analysis. Values exceeding physiologically plausible levels, or 4 SDs from the mean, were excluded as outliers.

### Statistical Analysis

We first determined cross‐sectional associations between BP and arterial stiffness in the larger population available at baseline and in the larger follow‐up population at the second follow‐up. Second, we described the evolution of these cross‐sectional associations with increasing age through stratification of the population. To directly assess longitudinal relationships in the smaller group of participants with ASI measured at baseline and at least 1 follow‐up visit, we determined associations between baseline measures and progression of each index during follow‐up, as percentage change of the dependent variable per annum. Finally, we determined the predictive value of baseline indices for the risk of progression to clinically defined hypertension or excessive arterial stiffness.

To assess factors affecting reliability of analyses, the consistency of measurements across follow‐up visits was first estimated by intraclass correlation coefficients, by linear correlation, and by Cronbach α. Mean differences were estimated and compared by paired *t* tests to assess for progression in indices over time. Participants were further stratified by the presence of a “notch” in the ASI waveform between the systolic and diastolic phase of the cardiac cycle. Mean differences between assessment centers at baseline and follow‐up were calculated, and compared by 1‐way ANOVA. To minimize between‐center variation, analyses were repeated for each index following standardization of values at each center to the distribution of values across all centers at that visit: standardizing each value by the number of SDs from the mean of its center to the mean and number of SDs of the whole population.

To determine the rate of progression of each index, participants were pooled across the 2 follow‐up visits as different participants returned at each follow‐up, with limited numbers assessed at both follow‐ups. Progression of each index of interest (ASI, SBP, DBP, and PP) was standardized as the percentage change per annum. For the limited participants attending both follow‐ups, the longer follow‐up was used to calculate the rate of progression.

Relationships between baseline and follow‐up indices at each visit, and between baseline and change between visits, were determined by general linear models, unadjusted and adjusted for age and sex; age, sex, and the baseline index and for age, sex, baseline index, and cardiovascular risk factors (cholesterol, current or ever‐smoking status, diabetes mellitus, BP). Models were repeated stratified by age older or younger than 60 years. Nonlinear interactions were assessed by addition of a squared term for age to univariate associations (between‐model comparison by ANOVA) and through stratification of results by quartiles of a second index. To determine the maximum point of nonlinear associations between BP and age, the maximum inflection point was identified by solving the third‐order fitted polynomial across deciles, with CIs determined by bootstrapping. The impact of regression dilution was assessed by nonparametric estimation of the regression dilution ratio, through division of the population into 5 evenly spaced bins of the index of interest: (mean in highest group at follow‐up−mean of lowest group at follow‐up)/(mean in highest group at baseline−mean in lowest group at baseline).^1^ Parametric adjustment was then performed by adjustment of the regression coefficient (or its logarithm in logistic regression) by the reliability ratio. The effect upon the proportion of variance explained by an association was estimated by disattenuation of the regression coefficients (r_xy_/(rr_x_×rr_y)_, where rr is the reliability ratio). Finally, multivariate adjustment for measurement variation was estimated via regression calibration.^22^

To assess the risk of progression to clinically defined forms of hypertension or excessive arterial stiffness, normotension was defined by baseline BP (<140/90 mm Hg), a reported diagnosis of hypertension or use of antihypertensive agents, with subdivision of the population into mixed hypertension or isolated systolic hypertension. Elevated ASI was defined as >10 m/s, consistent with European guidelines for carotid femoral pulse wave velocity.^10^ The effect of baseline measurements on the risk of transition between hypertensive states or to elevated arterial stiffness at either follow‐up was determined by logistic regression, with and without adjustment for cardiovascular risk factors or the effect of regression dilution.

Analyses were performed in R and SAS (SAS Institute).

## Results

The 169 742/502 536 UK Biobank participants with arterial stiffness (ASI) measured at baseline at 10 of 22 Biobank centers were largely similar to the remaining population (Table 1), with clinically insignificant differences for most measures, albeit statistically significant differences given the large population size. A total of 20 199 participants who returned at the first follow‐up visit were similar to the baseline population, while 31 418 participants who returned for the second follow‐up visit after a median of 8.5 years were younger when recruited, with lower SBP and body mass index, fewer smokers, and fewer having hypertension or diabetes mellitus. Only 1731 participants with ASI at baseline attended both follow‐ups and had ASI measured, while 13 210 participants had ASI and BP measured at baseline and at least 1 follow‐up (Figure S1).

**Table 1:** Baseline Characteristics of Patients Assessed at Baseline and Returning for the Second Follow‐Up Visit

Characteristic	All Participants (N=502 536)	ASI at Baseline (n=169 742)	*P* Value	Had ASI at Second Follow‐Up (n=31 418)	*P* Value vs Baseline
Baseline age, y	56.5 (8.1)	56.8 (8.2)	<0.0001	55 (7.5)	<0.0001
Men, No. (%)	229 134 (45.6)	77 730 (45.8)	0.045	15 495 (49.3)	<0.0001
Diabetes mellitus, No. (%)	26 402 (5.3)	9741 (5.7)	<0.0001	870 (2.8)	<0.0001
Smoking, No. (%)					
Current	52 979 (10.6)	17 081 (10.1)	<0.0001	2029 (6.5)	<0.0001
Ever	226 049 (45.1)	75 428 (44.4)	<0.0001	12 380 (39.4)	<0.0001
Hypertension, No. (%)	135 762 (27.1)	46 113 (27.2)	0.0564	6433 (20.5)	<0.0001
Previous events					
Stroke, No. (%)	7668 (1.5)	2491 (1.5)	0.0183	247 (0.8)	<0.0001
MI, No. (%)	11 608 (2.3)	3783 (2.2)	0.0074	375 (1.2)	<0.0001
Creatinine, μmol/L	72.3 (18.5)	72.6 (18.4)	<0.0001	72.5 (14.1)	0.6492
Cholesterol, mmol/L	5.7 (1.1)	5.7 (1.1)	0.0052	5.7 (1.1)	<0.0001
HDL, mmol/L	1.4 (0.4)	1.5 (0.4)	<0.0001	1.5 (0.4)	0.9559
LDL, mmol/L	3.6 (0.9)	3.5 (0.9)	<0.0001	3.6 (0.8)	<0.0001
Lipoprotein a, nmol/L	44.6 (49.2)	44.9 (49.1)	0.0287	44.1 (49)	0.0158
Weight, kg	78.1 (15.9)	78.2 (16)	<0.0001	77.4 (15.1)	<0.0001
BMI, kg/m^2^	27.4 (4.8)	27.5 (4.8)	0.0066	26.7 (4.3)	<0.0001
SBP, mm Hg	137.8 (18.7)	137.8 (18.6)	0.8892	135.4 (17.6)	<0.0001
DBP, mm Hg	82.2 (10.2)	82.1 (10.1)	<0.0001	81.5 (9.9)	<0.0001
PP, mm Hg	55.6 (13.6)	55.7 (13.6)	0.0007	53.8 (12.4)	<0.0001
ASI, m/s	9.3 (3.1)	9.3 (3.1)	···	9.2 (3)	0.0005
HbA_1c_, mmol/mol	36.1 (6.7)	36.3 (6.9)	<0.0001	35.1 (5.2)	<0.0001
Hypertension treatment, No. (%)	104 006 (26)	36 235 (27)	<0.0001	4415 (16.4)	<0.0001

Results are shown only for values at the baseline visit for all participants, participants with arterial stiffness index (ASI) measured at baseline, and participants with ASI measured at follow‐up. Values are reported as mean (SD) or number (percentage). Differences between groups are compared by *t* tests for continuous measures and chi‐square tests for discrete values. BMI indicates body mass index; DBP, diastolic blood pressure; HbA_1c_, glycated hemoglobin; HDL, high‐density lipoprotein; LDL, low‐density lipoprotein; MI, myocardial infarction; PP, pulse pressure; SBP, systolic blood pressure.

### Variability of BP and Arterial Stiffness Measures Over Time

To assess the effect of visit‐to‐visit variability on subsequent models, variability of measurement of ASI was significant across follow‐ups (α<0.4), with greater consistency (>0.6) for SBP or DBP between follow‐ups (Table 2). Variability was lower for measures between baseline and the first follow‐up than the second follow‐up. Variability of ASI improved slightly when only including participants with a “notch” in the arterial waveform at both baseline and follow‐up, despite a relatively low agreement between visits for the presence of a notch (Cohen κ: 0.25). In addition to the random variability in measures, there were systematic differences between visits, with a mean increase in SBP, PP, and ASI and a fall in DBP between visits (Table 1). Furthermore, there were systematic differences between centers for concurrently measured ASI and SBP, which was not evident for DBP and PP. Specifically, upon stratifying participants attending the first follow‐up visit (performed at 1 center) by the center they attended at baseline, there was greater variation between baseline centers for baseline SBP and ASI measures than for measures taken at the first follow‐up (Figure S2). There was also a systematically greater mean SBP and ASI at follow‐up 1 than either baseline or follow‐up 2 (Figure S2), including for the subset of participants attending all 3 visits. However, standardizing values to a global distribution only marginally increased the consistency of measures (Table 2).

**Table 2:** Variability of BP and Arterial Stiffness for Baseline Results vs the First or Second Follow‐Up Visit

Characteristic	Baseline to First Follow‐Up (4669)					Baseline to Second Follow‐Up (10 823)				
	ICC	CI	Cronbach α	Mean Increase	*R* ^2^	ICC	CI	Cronbach α	Mean Increase	*R* ^2^
Absolute values										
SBP	0.67	0.66–0.68	0.80	1.28	0.45	0.58	0.57–0.58	0.74	2.55	0.34
DBP	0.63	0.62–0.64	0.78	−1.41	0.41	0.51	0.50–0.52	0.70	−2.89	0.30
PP	0.66	0.65–0.67	0.81	2.68	0.46	0.52	0.51–0.53	0.74	5.41	0.35
Arterial stiffness	0.21	0.18–0.24	0.38	0.66	0.05	0.18	0.16–0.20	0.33	0.50	0.04
No notch	0.22	0.12–0.32	0.36	0.04*	0.05	0.12	0.03–0.20	0.23	−0.92	0.02
Notched	0.30	0.27–0.33	0.48	0.52	0.10	0.26	0.24–0.28	0.42	0.53	0.07
By center	0.23	0.20–0.25	0.38	0.13*	0.05	0.18	0.17–0.20	0.33	0.31	0.04

The intraclass correlation coefficient (ICC), its CI, and Cronbach α are reported, with the mean increase from baseline to follow‐up and the *R* ^2^ for a univariate linear regression. All analyses are significant at the *P*<0.001 level except for analyses with a *. BP indicates blood pressure; DBP, diastolic blood pressure; PP, pulse pressure; SBP, systolic blood pressure.

### Cross‐Sectional Associations Between Arterial Stiffness and BP

**Figure 1:**
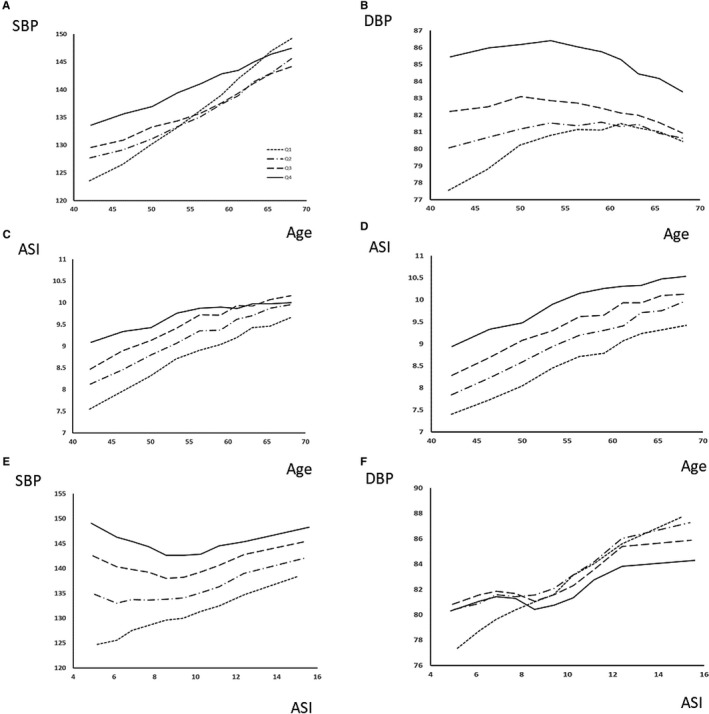
Interactions between age, blood pressure, and arterial stiffness index (ASI) at baseline. Values of mean systolic blood pressure (SBP) and diastolic blood pressure (DBP), pulse pressure (PP), and ASI are shown by deciles of age (**A** through **D**) or ASI (**E** and **F**), stratified by quartiles of ASI (**A**+**B**), SBP (**C**), DBP (**D**), or age (**E**+**F**).

At baseline, ASI and BP‐related measures were strongly associated with age, with the weakest linear association between age and DBP, and were greater in men, ever‐smokers, and with increased creatinine, weight, or body mass index (Table S1). However, despite an approximately linear relationship with age across all patients for baseline SBP, PP, and ASI, age was nonlinearly associated with DBP, with increasing DBP in lower deciles of age and falling DBP in later deciles (Figure 1), with addition of a squared term for age to the univariate association between age and DBP significantly increasing the amount of explained variance (univariate *r* ^2^=0.0015 to *r* ^2^=0.007, *P*<0.001; ANOVA). The maximum mean DBP occurred at age 55 years in men and 58.3 years in women.

**Figure 2:**
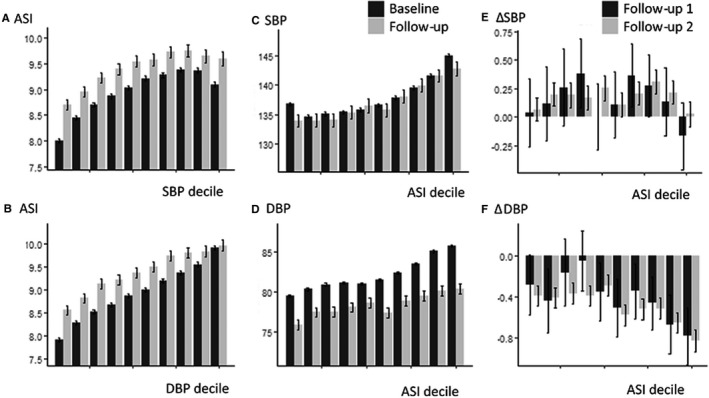
Mean absolute values of arterial stiffness index (ASI) and blood pressure at baseline and the second follow‐up (**A** through **D**), or change (Δ) per annum (**E** and **F**), by decile of baseline blood pressure or ASI. DBP indicates diastolic blood pressure; SBP, systolic blood pressure.

At baseline, ASI was positively correlated with all BP indices, with a stronger association with DBP than SBP, before and after adjustment for cardiovascular risk factors (Figure 2, Tables S1 and S2). There was a stronger association with DBP in a model including age, sex, cardiovascular risk factors, and both BP indices (β: SBP=−0.01, *P*<0.001; DBP=0.06, *P*<0.001). MBP and DBP were highly correlated (Table S1), and associations between ASI and DBP or MBP were similar but slightly weaker for MBP. There were modest but clinically significant increases in the magnitude of associations following adjustment for regression dilution by either nonparametric or parametric methods (Table S3), with the greatest increases in the magnitude of relationships between ASI and measures of BP (β unadjusted, 0.94; adjusted, 3.09) reflecting the greater variability of ASI.

The positive association between ASI with baseline SBP or PP significantly diminished after adjustment by age, sex, and cardiovascular risk factors, but remained significant for DBP, particularly in the highest deciles of arterial stiffness (Figure 1), with no effect of adjusting for heart rate (SBP: β=0.023, *P*=0.2; DBP: β=0.06, *P*<0.001). Associations were stronger for participants with notched ASI waveforms and when standardizing by center attended (Table S4), although the overall magnitude of the associations were limited by the greater variability of ASI. Adjustment for variability of ASI increased these associations and the proportion of explained variance (Table S3), with correction for variability of ASI increasing the relationship between ASI and baseline SBP from 0.94 mm Hg per m/s ASI to 3 mm Hg per m/s ASI, and the proportion of explained variance from 2% to 10%.

There was a complex interaction between age, ASI, and SBP or DBP (Figure 2). When split by quartiles of baseline ASI, baseline DBP was greater at all ages, but an increased ASI was associated with an earlier mean age of transition from a rising to a falling DBP (age at peak DBP by ASI quartile: highest quartile, 51.2 [95% CI, 49.9–52.3]; second quartile, 51.5 [95% CI, 50.0–53.3]; third quartile, 57.6 [95% CI, 55.5–59.1]; lowest quartile, 60.4 [95% CI, 59.6–61.3 years]). In contrast, although baseline SBP increased with age in all quartiles of ASI, this increase was steepest for patients at lower levels of initial ASI (Figure 2). As DBP remained low, this group had the greatest increase in PP across ages. Upon stratifying by age, there was a linear relationship between ASI and baseline SBP in the lowest quartile of age but a U‐shaped relationship in older quartiles. This did not reflect a different pulse‐waveform phenotype with age (Figure S3) but may be explained by the similar relationships with SBP variability across all visits with a U‐shaped relationship in older age groups between ASI and with SD of SBP or DBP (Figure S4).

### Longitudinal Relationships Between ASI and BP

In 13 761 participants with ASI measured at baseline and at least 1 follow‐up (13 210 with BP measurements), percentage change in ASI per annum at either follow‐up was negatively associated with increasing age, because of the strong baseline association between age and ASI and regression to the mean of participants with high ASI. Following adjustment for baseline ASI, increasing age was associated with an increase in ASI at follow‐up (Table S4). Similarly, following adjustment for baseline ASI, baseline SBP and DBP were associated with an increase in ASI across visits, before and after adjustment for age and risk factors (Table S4). There was no significant effect on the relationship between baseline SBP or DBP with increase in ASI across visits after adjustment for baseline heart rate (SBP, β=0.02 [*P*<0.001]; DBP, β=0.06 [*P*<0.001]). Furthermore, although use of hypertensive medications at baseline was associated with an increased risk of progression of ASI during follow‐up (β=0.44, *P*=0.008), use of antihypertensive medications was associated with reduced progression of ASI after adjustment for age, sex, and cardiovascular risk factors, but this was not significant (β=−0.38, *P*=0.16). In participants younger than 60 years, baseline DBP was more strongly associated with an increase in ASI at follow‐up than in participants older than 60 years (Table S5), but there was minimal difference in the association between baseline SBP and change in ASI in participants younger than or older than 60 years. Associations between progression of ASI were still slightly stronger for DBP than MBP such that DBP was the stronger predictor of increases in ASI during follow‐up. In contrast, ASI at baseline did not predict an increase in DBP at follow‐up but did predict an increase in SBP and PP, before and after adjustment for cardiovascular risk factors.

ASI and baseline BP were associated with a greater risk of transition to any hypertension, isolated systolic hypertension, or raised arterial stiffness (Table 3), with progression to hypertension being more strongly associated with a raised SBP or DBP at baseline, as expected. This association was increased after adjusting for regression dilution, with a 2.5‐fold increase in the risk of hypertension at follow‐up per 1.7 m/s ASI (the SD of ASI in participants with normotension, Table 3). Finally, both baseline ASI and baseline SBP or DBP were associated with transition to a raised ASI at follow‐up, with ASI being the strongest predictor after adjustment for regression dilution (Table S6).

**Table 3:** Risk of Progression to Hypertension or Increased ASI at Either Follow‐Up

	Normotension→Hypertension (n=13 635)		SDH→ISH (n=13 169)		Normotension→ISH (n=13 210)		ASI Transition (n=13 761)	
	OR	95% CI	OR	95% CI	OR	95% CI	OR	95% CI
Univariate								
ASI	1.36	1.28–1.44	0.98	0.90–1.07	1.31	1.23–1.39	1.22	1.17–1.27
SBP	2.59	2.49–2.69	1.40	1.33–1.48	2.59	2.47–2.71	1.25	1.19–1.3
DBP	1.82	1.76–1.88	0.74	0.70–0.78	1.39	1.35–1.45	1.21	1.16–1.27
Age	1.51	1.47–1.56	1.46	1.38–1.54	1.73	1.67–1.80	1.32	1.26–1.39
Female sex	1.17	1.15–1.19	1.04	1.01–1.08	1.10	1.07–1.12	1.12	1.09–1.16
Diabetes mellitus	1.05	1.02–1.09	1.00	0.94–1.07	1.04	1.00–1.08	1.04	0.98–1.10
Smoking current	1.02	0.99–1.06	0.98	0.92–1.04	0.97	0.93–1.01	1.04	0.98–1.09
Cholesterol	1.12	1.09–1.15	0.98	0.93–1.03	1.16	1.12–1.20	1.04	0.99–1.08
Partially adjusted								
ASI	1.18	1.11–1.26	0.92	0.84–1.01	1.11	1.04–1.20	1.16	1.11–1.22
SBP	2.49	2.39–2.60	1.31	1.24–1.39	2.45	2.34–2.58	1.13	1.08–1.19
DBP	1.88	1.82–1.95	0.76	0.71–0.80	1.44	1.39–1.50	1.17	1.12–1.23
Also adjusted for BP								
ASI	1.11	1.04–1.18	0.96	0.87–1.06	1.07	1.00–1.15	1.16	1.11–1.22
SBP	2.17	2.06–2.27	1.21	1.14–1.29	2.64	2.49–2.79	1.00	0.93–1.08
DBP	1.27	1.22–1.33	1.06	1.02–1.10	0.89	0.84–0.93	1.17	1.09–1.26

Adjusted for Visit‐to‐Visit Variability	Parametric	Nonparametric	Parametric	Nonparametric	Parametric	Nonparametric	Parametric	Nonparametric
ASI	2.61	2.54	0.94	0.95	2.31	2.25	2.78	1.84
SBP	4.79	3.34	2.32	1.53	4.78	3.34	1.35	1.32
DBP	2.52	2.10	0.33	0.69	1.67	1.51	1.30	1.26

Results are presented for progression from baseline normotension to any hypertension or isolated systolic hypertension (ISH), from mixed hypertension (systolic‐diastolic hypertension [SDH]) to ISH, and from low arterial stiffness index (ASI; <10 m/s) to high ASI (>10 m/s). Values are odds ratios (ORs) per SD of the dependent variable (by row) with 95% CIs. Univariate models are presented, with subsequent models presenting the OR for each dependent variable adjusted for age, sex, and cardiovascular risk factors (“partially adjusted”) or adjusted for age, sex, cardiovascular risk factors, and systolic blood pressure (SBP) and diastolic blood pressure (DBP) (“adjusted for BP”). Finally, univariate hazard ratios are reported adjusted for variability at follow‐up by parametric (Cronbach α) or nonparametric methods (regression dilution ratio). Numbers are reported for patients with blood pressure (BP) data at baseline and follow‐up and ASI at baseline.

## Discussion

In nearly 170 000 people, including more than 13 000 participants with repeated measurements over 4 to 9 years, there were strong associations between baseline SBP and DBP with arterial stiffness, with stronger associations for DBP than SBP. Midlife DBP was the strongest predictor of increases in arterial stiffness over time, while greater arterial stiffness was associated with increases in SBP. However, arterial stiffness was associated with an earlier age of transition to a falling DBP and was associated with the risk of transition to hypertension or elevated arterial stiffness during follow‐up.

The strong associations between arterial stiffness, hypertension,^6^ a consistently rising SBP with age, and a parabolic relationship with DBP are well‐established.^23^, ^24^ The demonstrated stronger relationship with future arterial stiffness for midlife steady components of BP contrasts with the prognostic importance of pulsatile BP components (isolated SBP, PP) in the elderly.^24^, ^25^, ^26^ However, if both the midlife risk associated with increased DBP and the late‐life increase in pulsatile pressures are both ultimately caused by midlife hypertension, then principally targeting late‐life pulsatile BP is suboptimal. However, there have been relatively few studies directly measuring the associations between midlife BP and progression of arterial stiffness over prolonged periods,^9^, ^12^, ^13^, ^14^ and none that we are aware of that directly compare midlife DBP with SBP and none that adequately assessed the effect of arterial stiffness on the age of transition to late‐life hypertensive phenotypes.^27^, ^28^ Although a number of interventions to reduce arterial stiffness have been examined,^29^, ^30^, ^31^ optimal prevention of late‐life harms may therefore depend on treating midlife factors that lead to arterial stiffening, before the transition to late‐life phenotypes.

In UK Biobank, arterial stiffness is associated with increased BP at all ages, with any hypertension predicting progression of arterial stiffness in the future, consistent with limited results from smaller studies.^6^, ^9^ However, baseline DBP was the strongest predictor of progression of arterial stiffness, with increased stiffness associated with an early transition to a fall in DBP, supporting the control of midlife DBP to prevent later arterial stiffening. Although there were similar associations for mean BP, the associations remained stronger for DBP, and DBP was therefore preferable as a covariate in adjusted models. The stronger relationship between midlife DBP and progression of arterial stiffness reflects the steady component of BP that may drive maladaptive large vessel changes with increases in collagen and calcification and a reduction in elastin. Alternatively, elevated midlife DBP may reflect distal vasoconstriction, increasing systemic resistance caused by either metabolic factors (smoking, obesity) or a primary small vessel arteriopathy, increasing wave reflection and secondary hemodynamic stress on large vessels.

The apparent linear relationship between baseline arterial stiffness and the future increase in SBP was relatively weak, particularly after adjustment for age or baseline SBP, but this partly reflected the nonlinear relationship between SBP and ASI in older age groups. In this group, there was a U‐shaped relationship between ASI and SBP associated with increased BP variability, implicating factors independent of ASI that result in an increase in SBP, eg, caused by white‐coat hypertension^32^ or age‐related baroreceptor dysfunction. Alternatively, it is possible that calculation of ASI, which utilizes the morphology of the waveform rather than direct measurement of the pulse wave transit time, may have underestimated arterial stiffness in some elderly patients with increased SBP, reflecting previous reports of suboptimal correlation between ASI and PWV.^21^ However, the mean waveform morphology across ASI tertiles was consistent across ages, suggesting that the variable increase in SBP at the second follow‐up reflected direct effects on SBP, rather than a misclassification of arterial stiffness (Figure S3).

A significant limitation of arterial stiffness, as measured in UK Biobank, is the greater variability of ASI compared with previous reports of the variability of arterial stiffness,^21^, ^33^ and compared with the variability of BP measurements in UK Biobank. This partly reflects the use of the PulseTrace device. Although this device was simple to apply in a large population and is correlated with arterial stiffness, it is less reproducible and only partly reflects large artery stiffness rather than being a direct measurement. This both reduces the power of the analyses to detect a significant effect of ASI and increases the possibility that the associations between demographic indices or BP with ASI and change in ASI may be caused by physiological factors other than arterial stiffness. However, the findings are consistent with previous reports of relationships between arterial stiffness, age, and BP,^9^ although these studies did not assess progression in such a large population or the effect of stiffness on age of transition of DBP. In addition, it reduces the estimated effect size for associations between ASI and clinical outcomes, other hemodynamic indices, or progression to hypertension in this cohort. Finally, because of the reliability ratio of ASI being <0.5 and its strong covariance with BP, there is an inherent limit to the reliability of conclusions that aim to adjust for measurement variation, which can be viewed as indicative only.^22^ Nonetheless, these analyses were consistent with the overall findings, biologically plausible, and demonstrate that conclusions reached without assessing the likely impact of measurement error are likely to be even more misleading, even if the absolute magnitude of adjusted associations remains uncertain. This also demonstrates the need to adjust for differential measurement error in epidemiological studies, and in UK Biobank in particular.

### Study Limitations

There are limitations to this analysis. The UK Biobank population is a community‐selected population of patients, resulting in ascertainment bias and a healthier population than the general population.^20^, ^34^ This is particularly pronounced for individuals reattending for magnetic resonance imaging at the second follow‐up. This limits external applicability to the whole population, but, given the large size of the study and internal consistency of the demonstrated associations, the effect of the relationships between BP and ASI are likely to be similar for the majority of individuals. In addition, there were systematic differences in UK Biobank between centers. This resulted in a center‐specific elevation in ASI and SBP at the first follow‐up. This may reflect a center‐specific factor resulting in temporary rather than sustained elevations in SBP and ASI, such as environmental causes of sympathetic activation at that center compared with other centers. However, standardizing results according to center attended, or including the center as a random effects variable, had no significant impact on the conclusions. Finally, the apparent strength of linear associations was often small, albeit with high significance levels because of the large size of the population. However, we have reported an *r* ^2^ change for most indices compared with a baseline model, producing a lower apparent strength of association than the *r* ^2^ for the whole model. Furthermore, the low proportion of variance explained was partly attributable to the greater variability of measurements, with significant improvements in the magnitude of association and the proportion of explained variance after adjustment for measurement variation. Finally, the true magnitude of differences is clinically significant despite relatively weak statistical associations. As such, any treatments affecting these indices at the population level are likely to be associated with significant population‐wide clinical benefits, beyond that implied by the statistical strength of association.

The principal implication of this work is that targeting hypertension to prevent arterial stiffening is likely to be effective at reducing detrimental clinical outcomes, but the optimal long‐term target is likely to be midlife BP, and DBP in particular. Studies in older populations may be better targeted at destiffening strategies or hemodynamically focused treatments that limit the distal effects of increased arterial stiffness.^35^ Furthermore, the apparent elevation in SBP in a subset of older individuals with low ASI needs further investigation to validate this finding in other populations with other methods of measuring arterial stiffness and to identify the underlying determinants and prognostic significance of elevated SBP in the absence of elevated arterial stiffness. Finally, characterization of the relationship between arterial stiffness and BP in UK Biobank is necessary to understand the effects of BP and arterial stiffness on end‐organ damage, including effects on cardiac and brain imaging measurements and clinical outcomes. In particular, future analyses need to allow for differential measurement error between variables and nonlinear relationships between age, BP, and arterial stiffness.

## Conclusions

Midlife DBP is the strongest predictor of progression of arterial stiffness, with elevated arterial stiffness associated with elevated SBP and DBP at all ages, an earlier transition from a rising to a falling DBP, and development of late‐life hypertensive phenotypes. Hypertension predicts progression of arterial stiffness, but elevated SBPs persist in a subgroup of elderly patients despite normal ASI. Finally, future epidemiological analyses, particularly in UK Biobank, must allow for the effects of the variability of measurements in predictive analyses of end‐organ damage and future cardiovascular events.

## Sources of Funding

This research is funded by an Alzheimer's Society grant (450‐AS‐PG‐18‐018). A.W. is also funded by a Wellcome Trust Clinical Research Career Development Fellowship (206589/Z/17/Z) and British Heart Foundation Project grant (PG/16/38/32080).

## Disclosures

None.

## Supporting information


**Table S1.** Associations Between Blood Pressure and Arterial Stiffness at Baseline With Demographic Features
**Table S2.** Associations Between Blood Pressure and Arterial Stiffness at Follow‐Up 2, With Demographic Features and ASI or Blood Pressure at Baseline
**Table S3.** Effect of Adjustment of Baseline Associations Between Blood Pressure, Arterial Stiffness, and Demographic Characteristics, Adjusted for Measurement Variation Between Baseline and the Second Follow‐Up Visit
**Table S4.** Associations Between Measures of Blood Pressure and Arterial Stiffness at Baseline, With Percentage Change in These Indices Per Annum at Follow‐Up, Adjusted for Baseline Measures, Standardized by Center and Including Only Patients With Notched ASI Recordings at Baseline and Follow‐Up
**Table S5.** Associations Between Measures of Blood Pressure and Arterial Stiffness at Baseline, With Percentage Change in These Indices at Follow‐Up, Adjusted for Baseline Measures, Standardized by Center and Age at Baseline Younger or Older Than 60 Years
**Table S6.** Risk of Progression to Hypertension or Increased ASI at Either Follow‐Up
**Figure S1.** Flow chart of participants at each stage of the study.
**Figure S2.** Recorded blood pressure (BP) and arterial stiffness measures at baseline and follow‐up, stratified by baseline Biobank assessment center.
**Figure S3.** Average pulse waveforms by tertile of arterial stiffness index (ASI) at baseline across the whole population, stratified by quartile of age.
**Figure S4.** Relationship between baseline arterial stiffness index (ASI), age, and blood pressure variability at baseline.
