# Supplementary material for: Progression of Arterial Stiffness is Associated With Midlife Diastolic Blood Pressure and Transition to Late‐Life Hypertensive Phenotypes
Source: J Am Heart Assoc. 2020 Jan 4;9(1):e014547. doi: 10.1161/JAHA.119.014547 (PMC6988142; doi:10.1161/JAHA.119.014547)

SUPPLEMENTAL MATERIAL

Table S1. Associations between blood pressure and arterial stiffness at baseline with demographic features. Beta-coefficients and r^2 are shown for univariate general linear models. In models adjusted for age / sex or for age / sex / cardiovascular risk factors (diabetes, current smoking and cholesterol). The beta-coefficient for each independent variable (by row) with the dependent variable (by column) is given, with the R^2 squared change compared to the models including only age and sex; or age, sex and cardiovascular risk factors. Associations are reported for the population in whom arterial stiffness index (ASI) was recorded at baseline. SBP=systolic blood pressure; DBP = diastolic blood pressure, PP = pulse pressure. All associations are significant, except where indicated *p>0.05. n=169,742. ASI = arterial stiffness index; SBP = systolic blood pressure; DBP = diastolic blood pressure; PP = pulse pressure; MBP = mean blood pressure; HTN = hypertension; BMI = body mass index;

	**SBP**		**DBP**		**PP**		**ASI**	
	**β**	**ΔR^2**	**β**	**ΔR^2**	**β**	**ΔR^2**	**β**	**ΔR^2**
Univariate								
**Age**	0.75	0.106	0.05	0.002	0.7	0.174	0.08	0.043
**Female Sex**	3.96	0.022	2.38	0.027	1.59	0.007	0.85	0.038
**Diabetes**	0.94	0.002*	-2.34	0.001	3.22	0.005	-0.05	0.002*
**HTN**	12.91	0.094	5.52	0.058	7.33	0.058	0.7	0.01
**Smoking Base**	-2.75	0.002	-0.65	0	-2.09	0.002	0.7	0.005
**Ever Smoked**	1	0.001	0.07	0*	0.93	0.001	0.61	0.01
**Creatinine**	0.06	0.004	0.03	0.004	0.03	0.002	0.02	0.009
**Cholesterol**	1.72	0.011	1.16	0.017	0.55	0.002	0	0*
**BMI**	0.73	0.035	0.59	0.077	0.14	0.003	0.07	0.013
**Weight**	0.2	0.03	0.18	0.081	0.02	0.001	0.03	0.03
**SBP**			0.38	0.488	0.62	0.715	0.03	0.024
**DBP**	1.28	0.488			0.28	0.045	0.06	0.034
**MBP**	1.42	0.837	0.79	0.863	0.46	0.149	0.05	0.034
**PP**	1.16	0.715	0.16	0.045			0.02	0.006
**ASI**	0.94	0.024	0.6	0.034	0.34	0.006		
+ Age / Sex								
**SBP**			0.41	0.502	0.59	0.56	0.01	0.004
**DBP**	1.25	0.452			0.25	0.035	0.05	0.022
**MBP**	1.38	0.734	0.81	0.855	0.41	0.108	0.03	0.014
**PP**	1.17	0.596	0.17	0.041			-0.01	0.001
**ASI**	0.4	0.007	0.52	0.02	-0.11	0.005		
+ CV RFs								
**SBP**			0.4	0.454	0.60	0.544	0.01	0.004
**DBP**	1.21	0.402			0.21	0.026	0.05	0.021
**MBP**	1.38	0.678	0.81	0.795	0.40	0.093	0.03	0.013
**PP**	1.13	0.561	0.13	0.056			-0.01	0.003
**ASI**	0.32	0.026	0.48	0.049	-0.15	0.01		

Dependent variables are shown in each column, with independent variables listed by row.

Table S2. Associations between blood pressure and arterial stiffness at follow-up 2, with demographic features and ASI or blood pressure at baseline. Beta-coefficients and r^2 are shown for univariate general linear models. In models adjusted for age / sex or for age / sex / cardiovascular risk factors (diabetes, current smoking and cholesterol) the beta-coefficient for each key index (row) with the dependent variable (by column) is given, with the R^2 squared change compared to the models including only age and sex; or age, sex and cardiovascular risk factors. Associations are reported for the population in whom arterial stiffness index (ASI) was recorded at baseline. SBP=systolic blood pressure; DBP = diastolic blood pressure; MBP = mean blood pressure; HTN = hypertension; PP = pulse pressure; BMI = body mass index; n=13761.

	**Measures at follow-up 2**							
	**SBP**		**DBP**		**PP**		**ASI**	
	**β**	**ΔR^2**	**β**	**ΔR^2**	**β**	**ΔR^2**	**β**	**ΔR^2**
Univariate								
**Age**	0.74	0.11	0.07	0	0.66	0.17	0.06	0.03
**Female**	4	0.02	2.36	0.03	1.65	0.01	1.23	0.1
**Diabetes**	1.66	0.001*	-2.09	0	3.7	0	-0.13	0.001*
**HTN**	13.11	0.1	6.04	0.07	7.02	0.06	0.59	0.01
**Smoking Base**	-3.5	0	-0.86	0	-2.64	0	0.59	0
**Ever Smoked**	0.73	0	0.02	0*	0.72	0	0.58	0.01
**Creatinine**	0.08	0.01	0.04	0.01	0.03	0	0.02	0.03
**Cholesterol**	1.74	0.01	1.13	0.02	0.6	0	-0.05	0
**BMI**	0.75	0.04	0.61	0.09	0.14	0	0.07	0.01
**Weight**	0.21	0.03	0.18	0.09	0.03	0	0.04	0.06
**Baseline SBP**			0.39	0.51	0.61	0.71	0.02	0.02
**Baseline DBP**	1.3	0.51			0.3	0.05	0.06	0.05
**Baseline MBP**	0.84	0.29	0.42	0.24	0.42	0.12	0.03	0.02
**Baseline PP**	1.18	0.71	0.18	0.05			0	0
**Baseline ASI**	0.83	0.02	0.78	0.05	0.06	0		
+ Age / Sex								
**SBP**			0.43	0.52	0.57	0.56	0	0
**DBP**	1.26	0.47			0.26	0.04	0.04	0.02
**MBP**	0.76	0.22	0.43	0.23	0.33	0.07	0.02	0.01
**PP**	1.18	0.59	0.18	0.05			-0.02	0.01
**ASI**	0.17	0	0.64	0.03	-0.46	0.01		
+ CV RFs								
**SBP**			0.41	0.46	0.59	0.54	0	0
**DBP**	1.22	0.41			0.22	0.03	0.04	0.02
**MBP**	0.74	0.19	0.42	0.20	0.31	0.06	0.02	0.01
**PP**	1.14	0.55	0.14	0.06			-0.02	0.01
**ASI**	0.08	0.02	0.59	0.05	-0.5	0.01		

Dependent variables are shown in each column, with independent variables listed by row.

Table S3. Effect of adjustment of baseline associations between blood pressure, arterial stiffness and demographic characteristics, adjusted for measurement variation between baseline and the second follow up visit. Adjustment for measurement variation is derived from the internal variability estimated in patients with ASI measured at baseline and the second follow-up visit. The beta-coefficient for the general linear model is reported for univariate associations, adjusted non-parametrically (see methods) or parametrically by Cronbach's alpha. Differences in R^2 values are determined by dis-attenuation. Multivariate adjustment for measurement variation is performed by multi-variate regression calibration. SBP=systolic blood pressure; DBP = diastolic blood pressure; MBP = mean blood pressure; HTN = hypertension; PP = pulse pressure; BMI = body mass index; n=169,742

	**SBP**			**DBP**			**PP**			**ASI**		
	β_{raw}	β_{α}	β_{np}	β_{raw}	β_{α}	β_{np}	β_{raw}	β_{α}	β_{np}	β_{raw}	β_{α}	β_{np}
Effect on β												
**Age**	0.75	0.75	0.75	0.05	0.05	0.05	0.70	0.70	0.70	0.08	0.08	0.08
**Creatinine**	0.06	0.08	0.07	0.03	0.04	0.03	0.03	0.04	0.03	0.02	0.03	0.02
**Cholesterol**	1.72	2.19	2.29	1.16	1.48	1.54	0.55	0.70	0.73			
**BMI**	0.73	0.82	0.76	0.59	0.66	0.62	0.14	0.16	0.15	0.07	0.08	0.07
**SBP**				0.38	0.53	0.48	0.62	0.87	0.79	0.03	0.04	0.04
**DBP**	1.28	1.85	1.59				0.28	0.40	0.35	0.06	0.09	0.07
**PP**	1.16	1.67	1.74	0.16	0.23	0.24				0.02	0.03	0.03
**ASI**	0.94	3.09	2.92	0.6	1.97	1.86	0.34	1.12	1.06			
Effect on r^2	r^2	r^2_{dis}		r^2	r^2_{dis}		r^2	r^2_{dis}		r^2	r^2_{dis}	
**Age**	0.106	0.144		0.002	0.003		0.174	0.236		0.043	0.130	
**Creatinine**	0.004	0.006		0.004	0.007		0.002	0.003		0.009	0.031	
**Cholesterol**	0.011	0.019		0.017	0.030		0.002	0.003		0.000	0.000	
**BMI**	0.035	0.050		0.077	0.115		0.003	0.004		0.013	0.042	
**SBP**				0.488	0.94		0.715	1.316		0.024	0.099	
**DBP**	0.488	0.94					0.045	0.087		0.034	0.146	
**PP**	0.715	1.308		0.045	0.086					0.006	0.024	
**ASI**	0.024	0.099		0.034	0.146		0.006	0.025				
+ Age / Sex	β	*p-val*		β	*p-val*		β	*p-val*		β	*p-val*	
**SBP**				0.72	<0.001		1.03	<0.001		0.02	<0.001	
**DBP**	2.42	<0.001					0.50	<0.001		0.09	<0.001	
**PP**	2.42	<0.001		0.34	<0.001					-0.01	<0.001	
**ASI**	2.10	<0.001		2.67	<0.001		-0.57	<0.001				
+ CV RFs												
**SBP**				0.72	<0.001		1.05	<0.001		0.02	<0.001	
**DBP**	2.51	<0.001					0.50	<0.001		0.09	<0.001	
**PP**	2.42	<0.001		0.34	<0.001					-0.01	<0.001	
**ASI**	2.15	<0.001		2.75	<0.001		-0.60	<0.001				

Dependent variables are shown in each column, with independent variables listed by row. Results are reported as β coefficients and r^2 for univariate models, and adjusted for age and sex or age, sex and CV risk factors (diabetes, current smoking and cholesterol)

Table S4. Associations between measures of blood pressure and arterial stiffness at baseline, with percentage change in these indices per annum at follow-up, adjusted for baseline measures, standardised by centre and including only patients with notched ASI recordings at baseline and follow-up. Beta-coefficients and r^2 are shown for univariate general linear models. In models adjusted for age / sex or for age / sex / cardiovascular risk factors (CV RFs = diabetes, current smoking and cholesterol) the beta-coefficient for each key index (row) with the dependent variable (by column) is given, with R^2 squared change compared to the models including only age and sex; or age, sex and CV RFs. Associations are reported for those with arterial stiffness index (ASI) was recorded at baseline. SBP=systolic blood pressure; DBP = diastolic blood pressure; MBP = mean blood pressure; HTN = hypertension; PP = pulse pressure; BMI = body mass index; n=13761

	**Percentage Change in Indices per annum at either follow-up**							
	**SBP**		**DBP**		**PP**		**ASI**	
**Baseline Indices**	**β**	**ΔR²**	**β**	**ΔR²**	**β**	**ΔR²**	**β**	**ΔR²**
Univariate								
**Age**	0.02	0.011	-0.02	0.01	0.10	0.044	0.03	0.001
**Female**	0.2	0.007	0.15	0.004	0.27	0.003	1.19	0.015
**Diabetes**	-0.2	0*	-0.15	0*	-0.64	0.001*	1.08	0*
**HTN**	0.3	0.004	0.12	0*	0.59	0.004	0.53	0.001
**Smoking Base**	0.07	0*	0.17	0*	-0.03	0*	0.16	0*
**Ever Smoked**	0.11	0.001	-0.01	0*	0.3	0.002	0.3	0*
**Creatinine**	0.01	0.004	0.01	0.001	0.01	0.004	0.03	0.003
**Cholesterol**	-0.02	0*	-0.04	-0.001*	0.01	0.002*	0.03	-0.002*
**BMI**	0.03	0.006	0.02	0.002	0.06	0.006	0.04	0.001
**Weight**	0.01	0.007	0.01	0.004	0.02	0.006	0.03	0.006
**SBP**	-0.04	0	-0.01	0.005	0.01	0.002	0.03	0.006
**DBP**	0	0*	-0.07	0	0.01	0.002	0.07	0.01
**MBP**	0.01	0.004	-0.03	0.005	-0.05	0	0.06	0.009
**PP**	0	0*	-0.01	0.005	-0.11	0	0.02	0.001
**ASI**	0.04	0.004	0.01	0*	0.1	0.007	-1.42	0
+ Age / Sex								
**SBP**	-0.04	0	-0.01	0.002	0.01	0.001	0.02	0.002
**DBP**	0.01	0*	-0.07	0	0.01	0.001	0.06	0.006
**MBP**	0.01	0*	-0.02	0.002	-0.06	0	0.05	0.006
**PP**	-0.01	0*	-0.01	0.002	-0.13	0	0	0*
**ASI**	0.02	0.001	0.01	0*	0.03	0*	-1.53	0
+ CV RFs								
**SBP**	-0.05	-0.001	-0.01	0	0.01	0.002*	0.02	-0.001
**DBP**	0.01	-0.001*	-0.07	-0.002	0.01	0.002*	0.06	0.003
**MBP**	0.01	0.001	-0.02	0.001	-0.058	0.001	0.05	0.005
**PP**	-0.01	-0.001*	-0.01	0	-0.13	0.002	0	-0.002*
**ASI**	0.01	-0.001*	0.01	-0.002*	0.02	0.002*	-1.54	-0.002
+ SBP + DBP								
**PP**	0	-0.001*	-0.01	-0.002	-0.14	0.001	-0.07	-0.003
**ASI**	0.01	-0.001*	0	-0.003*	0.02	0.001*	-1.57	-0.003

Dependent variables are shown in each column, with independent variables listed by row.

Table S5. Associations between measures of blood pressure and arterial stiffness at baseline, with percentage change in these indices at follow-up, adjusted for baseline measures, standardised by centre and by age at baseline less than or greater than 60 years. Beta-coefficients and r^2 are shown for univariate general linear models. In models adjusted for age / sex or for age / sex / cardiovascular risk factors (diabetes, current smoking and cholesterol) the beta-coefficient for each key index (row) with the dependent variable (by column) is given, with the R^2 squared change compared to the models including only age and sex; or age, sex and cardiovascular risk factors. Associations are reported for the population in whom arterial stiffness index (ASI) was recorded at baseline. SBP=systolic blood pressure; DBP = diastolic blood pressure; MBP = mean blood pressure; PP = pulse pressure; ASI = arterial stiffness index; All associations were significant except where indicated: * = p>0.05

	Percentage change in indices per annum at either follow-up							
	SBP		DBP		PP		ASI	
**Baseline Indices**	**β**	**ΔR^2**	**β**	**ΔR^2**	**β**	**ΔR^2**	**β**	**ΔR^2**
Age <60								
Univariate								
**Age**	0.03	0.01	-0.02	0.003	0.05	0.01	0.08	0.003
**Female**	0.16	0.005	0.19	0.008	0.06	0	0.63	0.003
**SBP**	-0.03	0	-0.004	0.001	-0.17	0.13	0.04	0.007
**DBP**	0.007	0.001	-0.07	0	0.34	0.13	0.07	0.009
**MBP**	0.01	0.001	-0.013	0.001	-0.04	0	0.06	0.009
**ASI**	0.05	0.009	0.018	-0.017	0.12	0.02	-1.43	0
Adjusted								
**SBP**	-0.04	0.002	-0.005	0.003	-0.19	0.15	0.03	0.001
**DBP**	0.008	0.003	-0.07	0.002	0.37	0.15	0.07	0.004
**MBP**	0.01	0.003	-0.014	0.003	-0.05	0	0.05	0.003
**ASI**	0.03	0.001	0.01*	-0.017	0.10	0.007	-1.50	-0.002
Age >60								
Univariate								
**Age**	0.01	0.0003	-0.02	0.001	-0.02	0	0.17	0.003
**Female Sex**	0.07	0.001	0.07	0.001	0.19	0.002	0.93	0.007
**SBP**	-0.04	0	-0.01	0.005	-0.13	0.12	0.03	0.005
**DBP**	-0.013	0.003	-0.07	0	0.27	0.11	0.04	0.002
**MBP**	-0.019	0.002	-0.03	0.004	-0.07	0	0.05	0.004
**ASI**	0.01*	0.002	-0.008	-0.001	0.08	0	-1.14	0
Adjusted								
**SBP**	-0.05	0.005	-0.01	0.009	-0.14	0.11	0.03	0.007
**DBP**	-0.015	0.008	-0.08	0.004	0.27	0.11	0.04	0.005
**MBP**	-0.022	0.007	-0.03	0.008	-0.08	0.003	0.04	0.007
**ASI**	0.004*	0.01	-0.01*	0.006	0.07	0.004	-1.46	0.004

Dependent variables are shown in each column, with independent variables listed by row.

Table S6. Risk of progression to hypertension or increased arterial stiffness index (ASI) at either follow-up. Results are presented for progression from normotension (norm) to any hypertension (HTN), mixed hypertension (SDH) to isolated systolic hypertension (ISH) or from low ASI (<10m/s) to high ASI (>10m/s). Values are odds ratios (OR) for logistic regression and 95% confidence intervals (CI), unadjusted and adjusted for variability, by non-parametric (NP) or parametric (Para) methods. . SBP=systolic blood pressure; DBP = diastolic blood pressure; BMI = body mass index

	**Norm → HTN**			**SDH → ISH**			**Norm → ISH**			**ASI Transition**		
	Unadj	Para	NP	Unadj	Para	NP	Unadj	Para	NP	Unadj	Para	NP
Unadjusted												
ASI	1.36	2.60	2.53	0.98	0.94	0.95	1.30	2.29	2.23	1.22	2.78	1.84
SBP	2.59	4.80	3.35	1.40	2.32	1.53	2.58	4.77	3.33	1.25	1.35	1.32
DBP	1.81	2.49	2.09	0.74	0.33	0.69	1.39	1.67	1.51	1.21	1.3	1.26
Cholesterol	1.12	1.14	1.15	0.98	0.97	0.97	1.16	1.20	1.21	1.04	1.04	1.05
Creatinine	1.18	1.23	1.21	1.00	1.00	1.00	1.07	1.09	1.08	1.09	1.11	1.1
BMI	1.38	1.43	1.40	0.94	0.93	0.93	1.18	1.20	1.19	1.05	1.05	1.05
Weight	1.35	1.37	1.36	0.94	0.93	0.94	1.13	1.14	1.13	1.11	1.12	1.11

Figure S1. Flow chart of participants at each stage of the study. The key analyses (in yellow) are performed for cross-sectional analyses at baseline in individuals in whom ASI was measured, and for rate of progression of indices by pooling individuals from both follow-ups, including participants with arterial stiffness index (ASI) measured at baseline and at a minimum of one follow-up. Most participants had blood pressure measured at baseline, but only a proportion had ASI measured (at specific centres). Different participants were followed up at each follow-up visit, with only a limited number who had ASI measured at baseline followed up at any follow-up, and a very limited number followed up at both follow-ups. The numbers progressing directly from one group to another are shown adjacent to arrows, whilst the number in each group are shown within each box.

A)

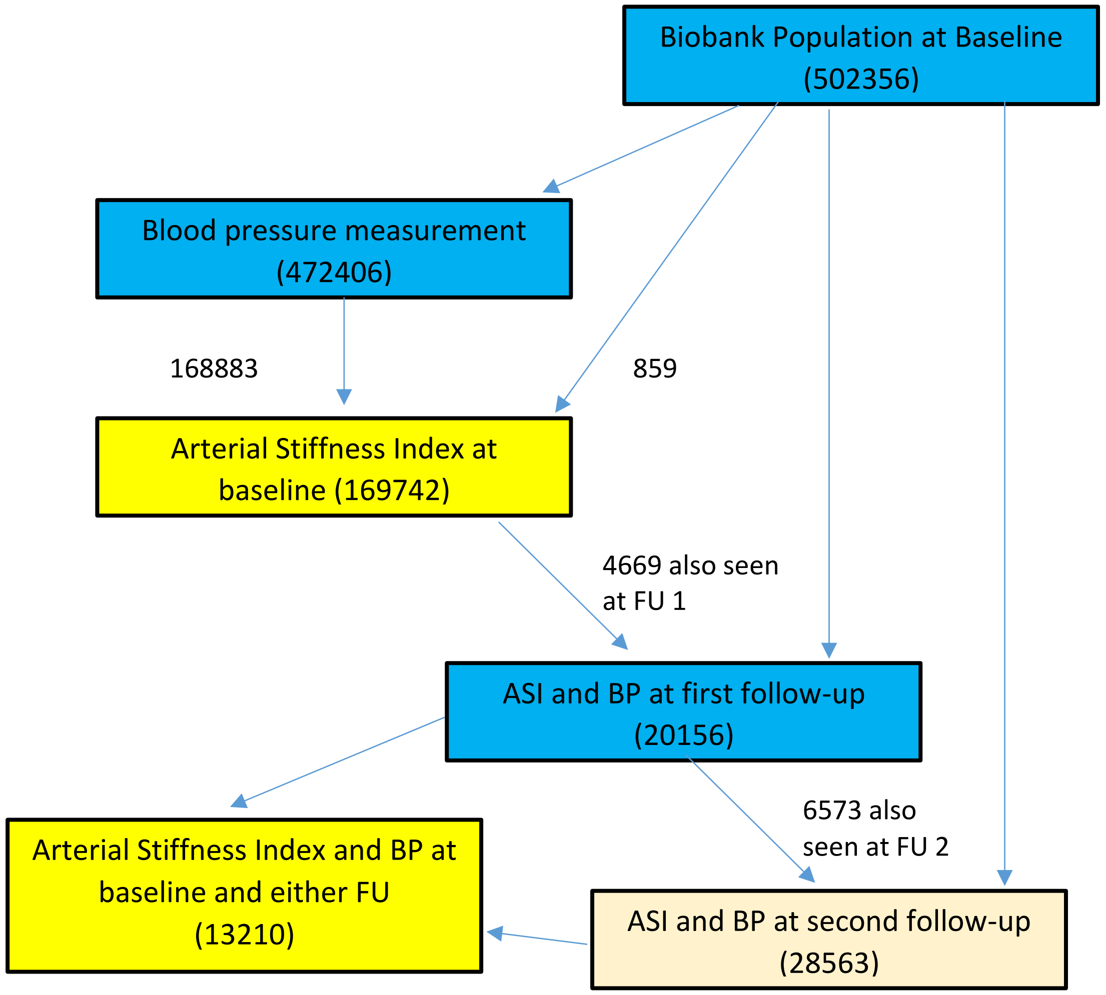

B) Statistical Analyses

Cross-sectional Relationships

- Performed at baseline and follow-up 2
- Associations between SBP, DBP, PP and ASI, univariate and adjusted for confounders (general linear models)
- Adjusted for visit-to-visit variability in predictors by regression calibration and regression dilution ratio
- Evolution of relationships between ASI, SBP and DBP with age determined by stratification into deciles of age, and quartiles of BP indices

Longitudinal Relationships

- In population with ASI and BP at baseline and either follow up
- Indices standardised by % change per annum
- Associations between baseline SBP, DBP, PP and ASI and progression of these indices determined for univariate associations and adjusted for confounders (general linear models)
- Risk of transition to hypertensive phenotypes (mixed hypertension, isolated systolic hypertension, diastolic hypertension) or excess ASI determined by logistic regression

Figure S2. Recorded BP and arterial stiffness measures at baseline and follow up, stratified by baseline Biobank assessment centre. Variation in mean indices at each centre is shown for mean ASI (A+B), DBP (C), SBP (D) and pulse pressure (E) at baseline, first follow-up and second follow-up, stratified by the baseline centre attended. Significant variation between centres is seen for ASI and SBP, with greater between-centre variation for measures taken at baseline than measures taken at follow-up for both SBP and ASI. Differences between visits and across follow-ups were more consistent for DBP and PP. SBP=systolic blood pressure; DBP = diastolic blood pressure; MBP = mean blood pressure; PP = pulse pressure; ASI = arterial stiffness index; FU = follow-up.

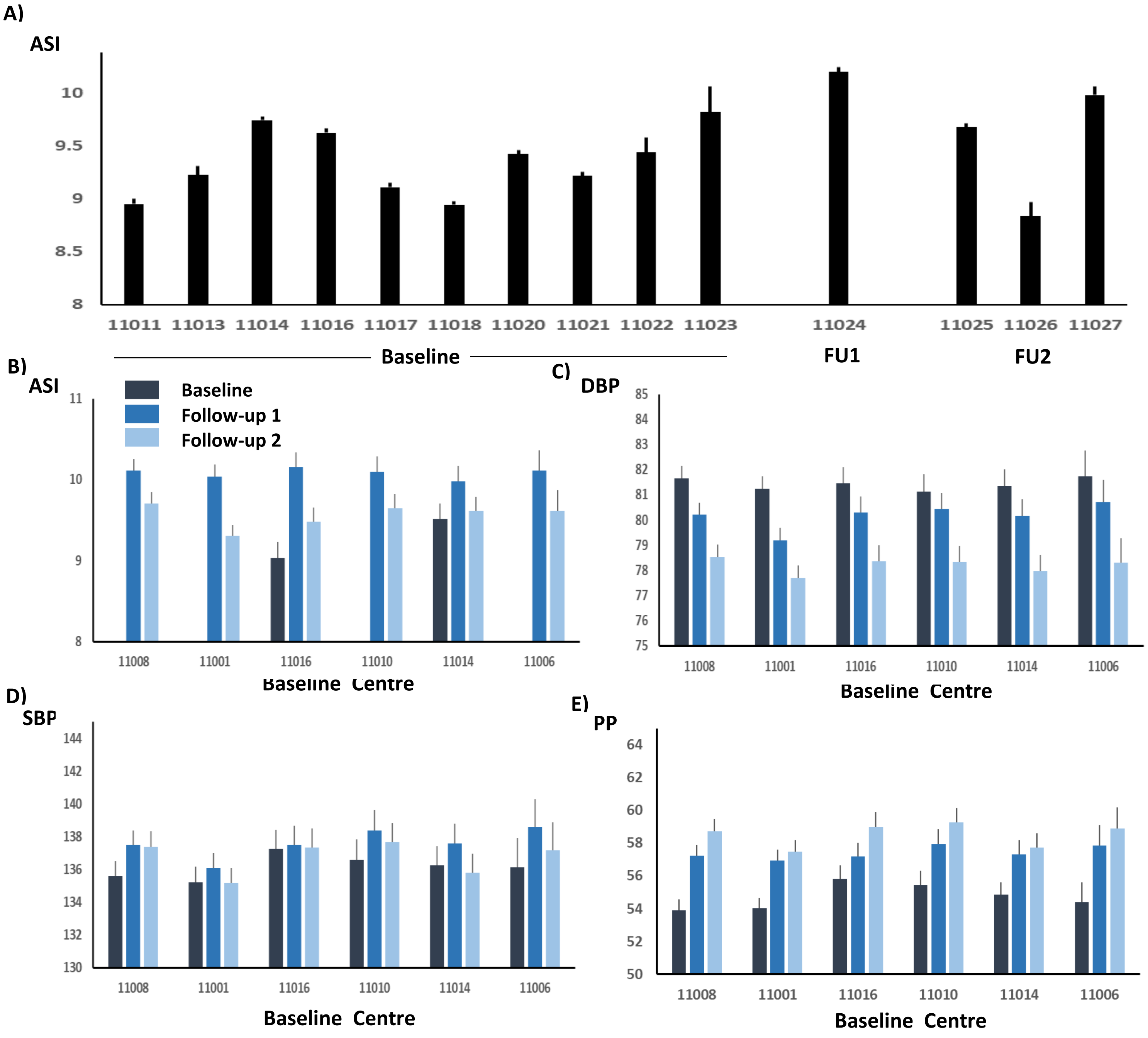

Figure S3. Average pulse waveforms by tertile of Arterial stiffness index (ASI) at baseline across the whole population, stratified by quartile of age.

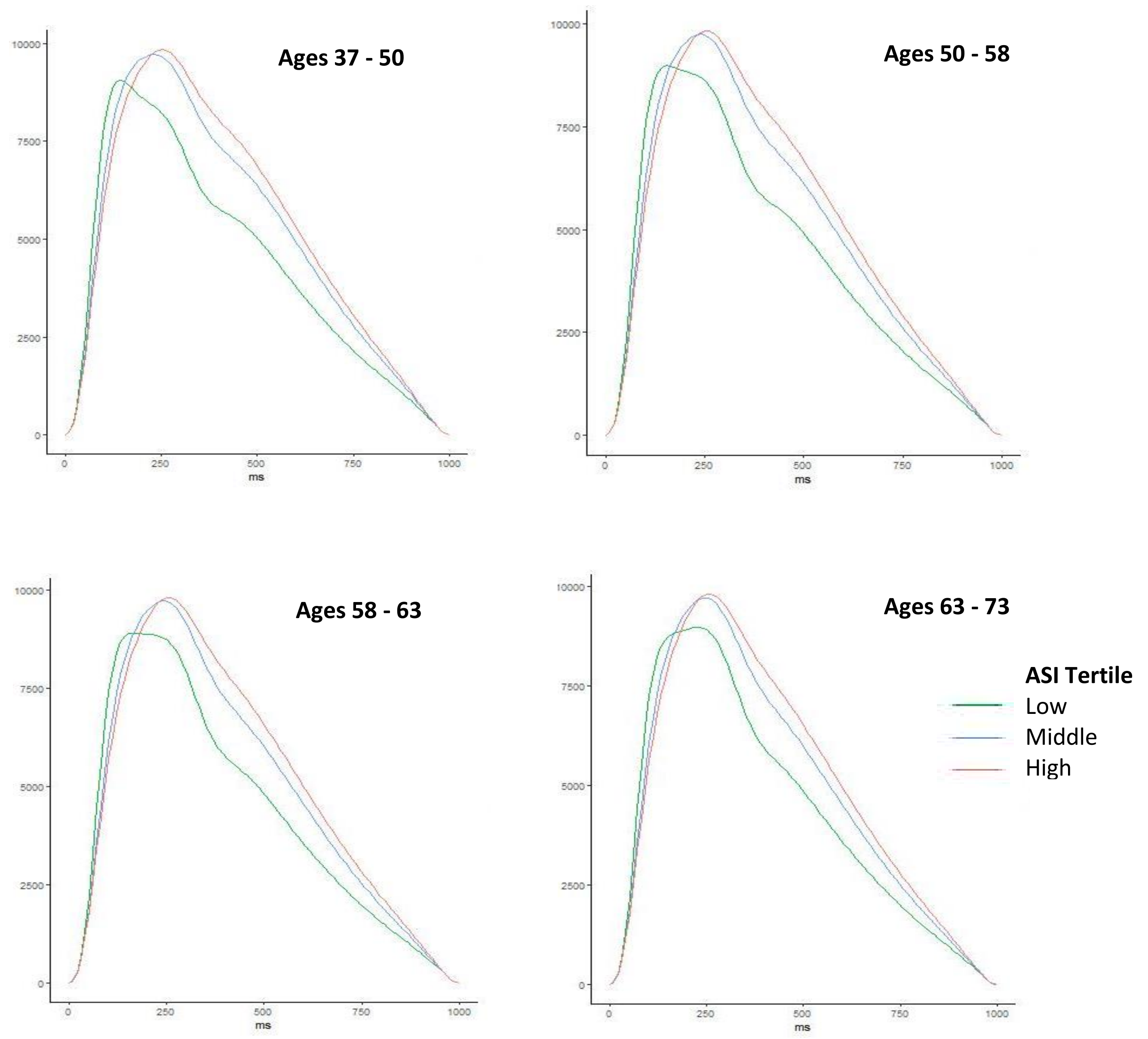

Figure S4. Relationship between baseline arterial stiffness index (ASI), age and blood pressure variability at baseline. ASI is divided into deciles, and stratified by quartiles of age, plotted against standard deviation of systolic BP (SBP-SD) or diastolic BP (DBP-SD) or their coefficients of variation (CV)

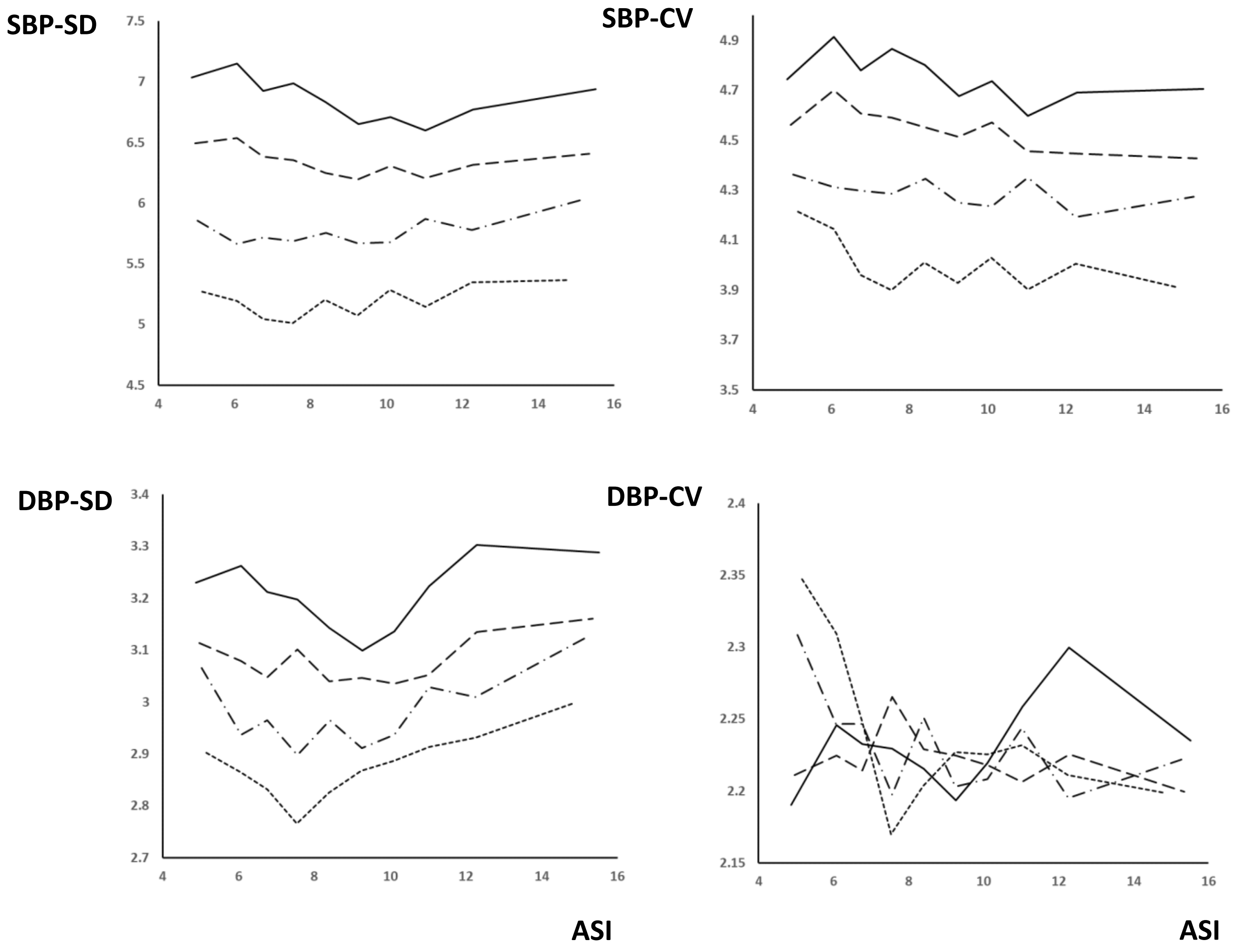

Supplement: Supplementary file 1 — Table S1. Associations Between Blood Pressure and Arterial Stiffness at Baseline With Demographic Features Table S2. Associations Between Blood Pressure and Arterial Stiffness at Follow‐Up 2, With Demographic Features and ASI or Blood Pressure at Baseline Table S3. Effect of Adjustment of Baseline Associations Between Blood Pressure, Arterial Stiffness, and Demographic Characteristics, Adjusted for Measurement Variation Between Baseline and the Second Follow‐Up Visit Table S4. Associations Between Measures of Blood Pressure and Arterial Stiffness at Baseline, With Percentage Change in These Indices Per Annum at Follow‐Up, Adjusted for Baseline Measures, Standardized by Center and Including Only Patients With Notched ASI Recordings at Baseline and Follow‐Up Table S5. Associations Between Measures of Blood Pressure and Arterial Stiffness at Baseline, With Percentage Change in These Indices at Follow‐Up, Adjusted for Baseline Measures, Standardized by Center and Age at Baseline Younger or Older Than 60 Years Table S6. Risk of Progression to Hypertension or Increased ASI at Either Follow‐Up Figure S1. Flow chart of participants at each stage of the study. Figure S2. Recorded blood pressure (BP) and arterial stiffness measures at baseline and follow‐up, stratified by baseline Biobank assessment center. Figure S3. Average pulse waveforms by tertile of arterial stiffness index (ASI) at baseline across the whole population, stratified by quartile of age. Figure S4. Relationship between baseline arterial stiffness index (ASI), age, and blood pressure variability at baseline. [file JAH3-9-e014547-s001.pdf]
